# Zur evaluierenden Audiometrie nach Cochlea-Implantat-Versorgung

**DOI:** 10.1007/s00106-023-01316-8

**Published:** 2023-07-14

**Authors:** Oliver C. Dziemba, Stephan Merz, Thomas Hocke

**Affiliations:** 1 Klinik und Poliklinik für Hals‑, Nasen‑, Ohrenkrankheiten, Kopf- und Halschirurgie, Universitätsmedizin Greifswald, Ferdinand-Sauerbruch-Str., 17475 Greifswald, Deutschland; 2 Merz Medizintechnik, Reutlingen, Deutschland; 3 Cochlear Deutschland GmbH & Co. KG, Hannover, Deutschland

**Keywords:** Prothesen und Implantate, Patientenspezifische Modellbildung, Lautheitsskalierung, Sprachaudiometrie, Computersimulation, Prostheses and implants, Patient-specific modeling, Loudness scaling, Speech audiometry, Computer simulation

## Abstract

**Hintergrund:**

Ein Hauptziel der Versorgung von Patient*innen mit Cochlea-Implantat (CI) ist die Verbesserung des Sprachverstehens. Einer der Zielparameter ist die Sprachverständlichkeit in Ruhe. Die Versorgungsergebnisse lassen jedoch eine sehr große Variabilität erkennen, welche bislang nur unzureichend erklärt werden konnte. Ziel dieser nichtinterventionellen retrospektiven Studie war die Aufklärung dieser Variabilität. Dies erfolgte anhand einer ausgewählten Population von Patient*innen, bei der die Ätiologie keinen negativen Einfluss auf die postoperative Sprachverständlichkeit erwarten ließ.

**Material und Methoden:**

Es wurden die audiometrischen Befunde der CI-Folgetherapie von 28 erwachsenen Patient*innen nach 6 Monaten CI-Erfahrung ausgewertet. Diese wurden in Relation zur präoperativen audiometrischen Untersuchung gesetzt und hinsichtlich eines unlängst publizierten Prädiktionsmodells für das postoperative Einsilberverstehen ausgewertet.

**Ergebnisse:**

Durch Einschluss der postoperativen Hörfeldskalierung und des Hörverlusts für Zahlen in das Modell lassen sich 55 % der Variabilität in den Versorgungsergebnissen bzgl. des Einsilberverstehens erklären.

**Schlussfolgerung:**

Die Ergebnisse dieser Studie legen nahe, dass ein Großteil der Ursachen für die Variabilität der Versorgungsergebnisse durch systematische postoperative audiometrische Kontrollen erfasst werden kann. Aus diesen Ergebnissen können sich unmittelbare Schlussfolgerungen für die Anpassungen der CI-Systeme ziehen lassen. Inwieweit diese jedoch von den einzelnen Patient*innen akzeptiert werden und somit zu einer Verbesserung der Befundlage führen, muss Gegenstand weiterer, möglichst prospektiver Studien sein.

## Abstract (en)

**Background:**

One of the main treatment goals in cochlear implant (CI) patients is to improve speech perception. One of the target parameters is speech intelligibility in quiet. However, treatment results show a high variability, which has not been sufficiently explained so far. The aim of this noninterventional retrospective study was to elucidate this variability using a selected population of patients in whom etiology was not expected to have a negative impact on postoperative speech intelligibility.

**Materials and methods:**

Audiometric findings of the CI follow-up of 28 adult patients after 6 months of CI experience were evaluated. These were related to the preoperative audiometric examination and evaluated with respect to a recently published predictive model for the postoperative monosyllabic score.

**Results:**

Inclusion of postoperative categorical loudness scaling and hearing loss for Freiburg numbers in the model explained 55% of the variability in fitting outcomes with respect to monosyllabic comprehension.

**Conclusion:**

The results of this study suggest that much of the cause of variability in fitting outcomes can be captured by systematic postoperative audiometric checks. Immediate conclusions for CI system adjustments may be drawn from these results. However, the extent to which these are accepted by individual patients and thus lead to an improvement in outcome must be subject to further study, preferably prospective.

## Einleitung

Die Versorgung mit einem Cochlea-Implantat (CI) ist eine Therapieoption für Patient*innen, die an höhergradiger Schallempfindungsschwerhörigkeit leiden und bei denen das CI im Vergleich zu anderen Therapieformen ein besseres Versorgungsergebnis erwarten lässt [5]. Bei der invasiven Therapieform des CI ist es daher von großer Bedeutung, die postoperativ erwartbare Sprachverständlichkeit bereits bei Indikationsstellung möglichst genau abschätzen zu können.

Am Anfang der CI-Versorgung waren die Möglichkeiten der präoperativen Differenzialdiagnostik limitiert, da die Indikation i. d. R. nur bei beidohriger, vollständiger funktioneller Taubheit gestellt wurde [8]. Auch wenn in den letzten Dekaden zunehmend Patient*innen mit funktionellem Restgehör mit einem CI versorgt wurden, verwundert es kaum, dass Prädiktionsstudien mit großen Fallzahlen [1, 17, 21] anamnestische, ätiologische und chirurgische Faktoren als stärkste Einflussgrößen identifiziert haben: Ein eventuell vorhandenes Restgehör spielte in diesen Arbeiten eine eher untergeordnete Rolle. Blamey et al. [1] identifizierten 5 Faktoren, die einen wesentlichen Einfluss auf die zu erwartende Sprachverständlichkeit haben:

- die Dauer der Ertaubung,
- das Alter bei Einsetzen einer hochgradigen Schwerhörigkeit,
- das Alter zum Zeitpunkt der CI-Versorgung,
- die Ätiologie und
- die Hörerfahrung mit CI.

Die Entwicklungen in Technik, Audiologie, Chirurgie und Rehabilitation [4, 7, 12, 15, 16, 19, 22–26, 29] führten im letzten Jahrzehnt zu besseren Versorgungsergebnissen. Infolgedessen wurden bei Hörgeräteträger*innen mit noch nutzbarer Sprachverständlichkeit eine CI-Indikation gestellt, deren Versorgungsergebnis das der Hörgeräteversorgung deutlich übertreffen konnte. Somit spielt die sehr genaue Vorhersage der absehbar zu erreichenden Sprachverständlichkeit eine gewichtige Rolle bei der Versorgung dieser Patient*innen. Bei erheblichem Resthörvermögen, hierzulande bis hin zu 60%iger monauraler Einsilberverständlichkeit bei 65 dB_SPL_ mit bestangepasstem Hörgerät, EV_65_(HG), gewinnen verlässliche Prognosen auf Basis präoperativer Befunde für Indikationsstellung, Beratung und Qualitätskontrolle verstärkt an Bedeutung [20, 28].

Hoppe et al. [18] zeigten, dass eine individuelle Prognose der erreichbaren Sprachverständlichkeit nach CI-Versorgung möglich ist. In dieser Studie wurde ein generalisiertes lineares Modell (GLM) angewendet. Dieses GLM basiert auf der präoperativ gemessenen, maximalen Einsilberverständlichkeit (mEV), der EV_65_(HG) und dem Lebensalter bei Implantation. Hoppe et al. [18] weisen auch auf die große Variabilität der Daten hin. Jedoch lassen sich trotz dieser Variabilität auch für den Einzelfall anwendbare, klinisch relevante Aussagen ableiten [18, 20]. So erreichen bzw. übertreffen 3 Viertel der untersuchten CI-Träger*innen die Vorhersage innerhalb eines Fensters von −12 Prozentpunkten (pp) [18]. Eine weitergehende und detaillierte Analyse hinsichtlich möglicher Ursachen für die gefundene Variabilität fand dort nicht statt. Hierfür bietet sich die Kombination des GLM mit den Ergebnissen einer Arbeit von Blamey et al. [1] an. Sie zeigten, dass bestimmte Ätiologien einen tendenziell negativen Einfluss auf das Versorgungsergebnis haben können. Würde man Patient*innen mit den dort beschriebenen negativ wirkenden Ätiologien aus der Modellbildung ausschließen, muss zwangsläufig der Teil der Variabilität, welcher sich durch extrinsische Faktoren erklären ließe, größer werden. Sollten also Patient*innen in dieser Population unterhalb der Vorhersage liegen, haben spezielle Aspekte der CI-Anpassung, der Rehabilitation und der Prozesskontrolle sicher einen stärkeren Einfluss: Die Differenzen zur Vorhersage werden sich weitgehend mit den Daten aus der klinisch-audiologischen Evaluation der Prozessoreinstellungen begründen lassen.

So ist es dann das Ziel dieser Arbeit, den Zusammenhang zwischen postoperativer Einsilberverständlichkeit und deren präoperativer Prognose zu untersuchen. Zur Analyse dieser Variabilität dienen weitere klinisch-audiologisch obligate Parameter, wie die Hörfeldskalierung und die Ermittlung des Hörverlusts für Zahlen. Obiger Argumentation folgend wurden Patient*innen mit einer Ätiologie, deren Einfluss ein unterdurchschnittliches Versorgungsergebnis erwarten lässt, ausgeschlossen. In der Folge tritt der Einfluss möglicher extrinsischer Faktoren in der Studienpopulation deutlicher hervor.

## Material und Methoden

### Einschlusskriterien und Probanden

**Figure 1:**
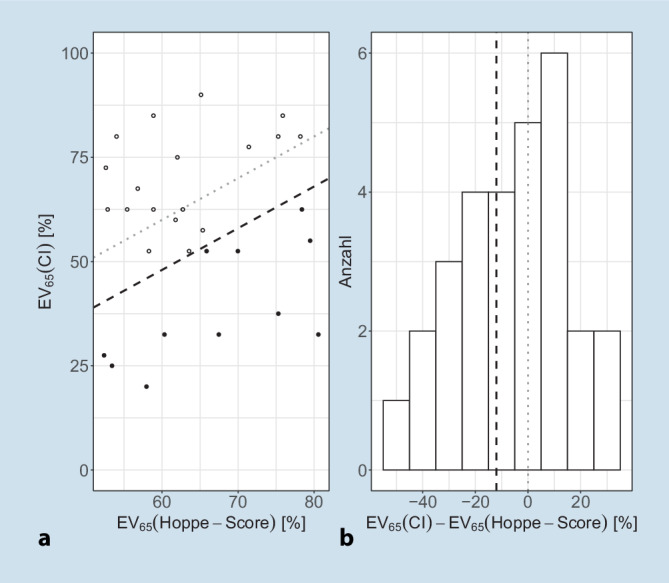

Die Einschlusskriterien zur retrospektiven Datenauswertung waren wie folgt definiert:

- Alter zum Zeitpunkt der CI-Versorgung mindestens 18 Jahre,
- Ätiologie, deren mittlerer Prozentrang nach Blamey et al. (Fig. 6; [1]) ≥ 0 % lag,
  - Hörsturz, genetisch, M. Menière, Otosklerose, unbekannt, akustisches Trauma, verschiedene,
- präoperative Daten vorhanden:
  - unversorgte mEV,
  - EV_65_(HG),
- audiometrische Befunde zum Zeitpunkt 6 Monate nach Erstanpassung vorhanden:
  - Hörverlust für Zahlwörter (HVZ),
  - Freiburger Einsilbertest in Ruhe im Freifeld bei 50/65/80 dB_SPL_,
  - monaurale Sprachverständlichkeitsschwelle L_50_ im Störschall mit dem Oldenburger Satztest (OLSA),
  - Hörfeldskalierung in den Frequenzen 250/500/1000/2000/4000 Hz,
- schriftliche Einwilligung der Patient*innen in die anonyme Datenverarbeitung der Daten aus der klinischen Routine.

Explizit ausgeschlossen wurden Fälle mit den Ätiologien Ototoxizität, Labyrinthitis, chronische Otitis media, Meningitis, Felsenbeinfraktur, Schwannome, auditorische Synaptopathie und Neuropathie, deren Versorgungsergebnis nach Blamey et al.[1] vergleichsweise unterdurchschnittlich bleiben.

In diese Studie wurden 29 CI-Versorgungen an 28 Patient*innen (12 männlich, 16 weiblich) eingeschlossen. Bei einer bilateral-sequenziellen CI-Versorgung konnten beide Seiten in die Auswertung eingeschlossen werden. Die Versorgungen gruppierten sich in 13 linksseitige und 16 rechtsseitige Versorgungen. Das mittlere Alter zum Zeitpunkt der CI-Versorgung betrug 59,3 Jahre (min. 30 Jahre, max. 81 Jahre). Eine Selektion nach CI-Hersteller erfolgte nicht. Die jeweilige Anzahl pro CI-Hersteller ist in Tab. 1 aufgelistet.

**Table 1:** 

Hersteller	Anzahl
Fa. Advanced Bionics (Stäfa, Schweiz)	3
Fa. Cochlear (Sydney, Australien)	19
Fa. MED-EL (Innsbruck, Österreich)	7

### Präoperative Vorhersage des Versorgungsergebnisses in Ruhe

Zur Abschätzung der individuellen Einsilberverständlichkeit mit CI in Ruhe bei 65 dB_SPL_, EV_65_(CI), nach einer Tragedauer von etwa 6 Monaten aus den präoperativen audiometrischen Daten erfolgte die Berechnung nach Gl. 1 [18]. Dieser Vorhersagewert soll im Weiteren als Hoppe-Score bezeichnet werden. Die nötigen Koeffizienten (β-Werte) sind in Tab. 2 gelistet.

1 $$\mathrm{EV}_{65}\left(\mathrm{CI}\right)\left[\mathrm{{\%}}\right]=\frac{100}{1+e^{-\left(\beta _{0}+\beta _{1}\cdot \mathrm{mEV}+\beta _{2}\cdot \text{Alter}+\beta _{3}\cdot \mathrm{EV}_{65}(\mathrm{HG})\right)}}$$

**Table 2:** 

Koeffizient	Wert	[β]
β_0_	0,84	–
β_1_	0,012	1/%
β_2_	−0,0094	1/Jahr
β_3_	0,0059	1/%

Positive β‑Werte bedeuten einen positiven Einfluss der korrespondierenden Variablen auf die Prognose und vice versa

### Audiometrische Messungen mit CI

Alle audiometrischen Messungen erfolgten in Räumen, welche den Anforderungen aus der Normreihe DIN EN ISO 8253 vollumfänglich genügten. An allen Messplätzen war das Audiometer MA55 (Fa. MAICO Diagnostics GmbH, Berlin, Deutschland) installiert. Die verwendeten Kopfhörer waren DT48 (Fa. beyerdynamic GmbH & Co. KG, Heilbronn, Deutschland) oder PD-95 (Fa. Holmberg GmbH & Co. KG, Berlin, Deutschland). Durch die regelmäßigen messtechnischen Kontrollen (MTK) und hörerabhängige Freifeldentzerrung können Ergebnisabweichungen zwischen den verwendeten Kopfhörern vernachlässigt werden.

Zur präoperativen Sprachaudiometrie mit Hörgerät im freien Schallfeld wurde der Lautsprecher 8020D (Fa. GENELEC®, Iisalmi, Finnland) verwendet. Alle postoperativen audiometrischen Messungen mit CI im freien Schallfeld erfolgten unter Verwendung des Lautsprechers LAB-251 (Fa. Westra Elektroakustik GmbH, Wertingen, Deutschland).

#### Sprachaudiometrie in Ruhe

Die Messungen der Sprachverständlichkeit in Ruhe erfolgte mit dem Freiburger Sprachtest [14] der sich aus einem Zahlen- und Einsilbertest zusammen setzt. Die präoperative Messung der mEV erfolgte seitengetrennt über Kopfhörer. Die mEV ergibt sich als prozentuales Maximum aus der gemessenen Diskriminationsfunktion des Freiburger Einsilbertests. Die EV_65_(HG) wurde mit dem Freiburger Einsilbertest im freien Schallfeld mit einer Entfernung zum Lautsprecher von 1 m und einer frontalen Schalleinfallsrichtung gemessen. Eventuell auftretende Überhöreffekte wurden durch die allgemein übliche Vertäubung über Kopfhörer unterbunden.

Im Rahmen der postoperativen Evaluation der Sprachaudiometrie in Ruhe erfolgte die Messung des HVZ und der Einsilberverständlichkeit für die Sprachschallpegel 50/65/80 dB_SPL_. Dieses Vorgehen ist an der Einrichtung des Erstautors klinischer Standard und wurde bereits mehrfach beschrieben [9, 11].

#### Hörfeldskalierung

Zur Darstellung der überschwelligen Dynamik mit CI kann eine Hörfeldskalierung nach DIN ISO 16832 [6] gemessen werden. Diese Hörfeldskalierung wurde mit dem Oldenburger Messprogramm OMA (HörTech gGmbH, Oldenburg, Deutschland) in der Version 1.5.5.0 adaptiv gemessen [2]. Wie in einer Arbeit von Dziemba et al. [11] beschrieben, können aus der Hörfeldskalierung durch Regression Kurven gleich lauter Hörempfindung für den gemessenen Frequenzbereich ermittelt werden. Für die Darstellung der Lautheitskategorien wurde eine 11-stufige Skala – sehr leise (5 CU, „categorial unit“, kategoriale Einheit), leise (15 CU), mittel (25 CU), laut (35 CU), sehr laut (45 CU) und zu laut (50 CU) – verwendet.

Um die Bestimmung der frequenzspezifischen Hörschwelle mit CI unabhängig von der Steilheit der Pegel-Lautheits-Funktion zu gestalten, wurde die Hörschwellenbestimmung aus der Hörfeldskalierung nach Rader et al. [25] angewendet.

Die Extraktion der Rohdaten aller Messungen erfolgte mit einem proprietären Softwaremodul der Fa. Merz Medizintechnik GmbH (Reutlingen, Deutschland).

#### Sprachaudiometrie im Störschall

Der OLSA ist ein Matrixtest nach dem Vorbild von Hagermann [13], der für deutsche Sprache adaptiert, optimiert und für Messungen im Störschall in einer Referenzsituation evaluiert wurde [30–32]. Mit dem OLSA können prozentuale Sprachverständlichkeitsschwellen (SVS) im Störschall adaptiv gemessen werden. Die adaptive Messung einer 50%-Sprachverständlichkeitsschwelle (L_50_) im Störschall erfolgt dabei durch Variation des Darbietungspegels eines Signalanteils (Sprache oder Störschall), wobei das jeweils andere Signal (Störschall oder Sprache) im Darbietungspegel fixiert bleibt [3]. Entsprechend eigener Untersuchungen [9] wurde bei monauraler Sprachaudiometrie im Störschall mit dem OLSA das Sprachsignal im Pegel bei 65 dB_SPL_ fixiert gehalten. Aus der Differenz von Sprachschallpegel und dem Pegel im L_50_ ergibt sich der maximal zulässige Störschallpegel an der SVS im Störschall als „acceptable noise level“ (ANL).

Die postoperative monaurale Messung des ANL mit CI erfolgte mit dem Oldenburger Messprogramm OMA (Fa. HörTech gGmbH, Oldenburg, Deutschland) in der Version 1.5.5.0. Die Methodik bei allen Messungen erfolgte entsprechend dem hausinternen Standard in Analogie zu Dziemba et al. [9].

## Ergebnisse

### Darstellung nach Hoppe et al. [18]

In Abb. 1 sind die EV_65_(CI) nach 6 Monaten Versorgungsdauer über der Prognose im Scatter-Plot (a) und Histogramm (b) nach Hoppe et al. [18] dargestellt. Die gestrichelten Linien zeigen die Winkelhalbierende (grau) und das erste Quartil bei −12 pp für die EV_65_(CI) aus Hoppe et al. [18] (schwarz) im Scatter-Plot und äquivalent im Histogramm.

Als Trennkriterium der Versorgungsergebnisse haben die Autoren das erste Quartil des Hoppe-Scores der Originalarbeit [18] verwendet. Somit teilt sich die Grundgesamtheit in 18 CI-Versorgungen, bei denen das prognostizierte Versorgungsergebnis als erreicht betrachtet werden kann (Gruppe 1). Bei 11 CI-Versorgungen wurde die prognostizierte Einsilberverständlichkeit um mehr als 12 pp unterschritten (Gruppe 2). Bemerkenswert ist hierbei, dass nach dieser dynamischen und individuellen Definition 4 Fälle mit offenem Sprachverstehen, EV_65_(CI) > 50 %, in die Gruppe 2, also als noch nicht erreichtes Versorgungsziel, eingeordnet werden müssen.

### Lautheitsskalierung

**Figure 2:**
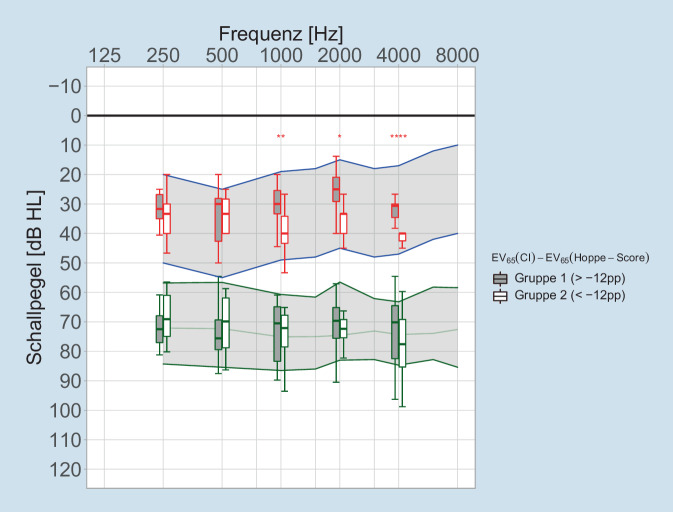

In Abb. 2 sind die Hörschwelle mit CI nach Rader et al. [25] und die Pegel gleich lauter Hörempfindung mit CI für die Lautheitskategorie „mittel“ (25 CU) für die Frequenzen 250/500/1000/2000/4000 Hz gruppiert nach Gruppe 1 und Gruppe 2 nach 6 Monaten Versorgungsdauer als Lage- und Streumaße dargestellt. Als von den Autoren postulierter Zielwert für die Pegel mittellauter Hörempfindung mit CI sind die Referenzwerte der Hörfeldskalierung Normalhörender aus der DIN ISO 16832 [6] als grün eingefasster und grau hinterlegter Bereich dargestellt.

Um die Auswirkungen unterschiedlicher schwellennaher Hörempfindungen auf die Sprachverständlichkeit darzustellen, wurde das Sprachpegelfeld für repräsentative normallaute deutsche Sprache (L_eq_ = 65 dB_SPL_) nach Steffens [27] als blau eingefasster und grau hinterlegter Bereich dargestellt.

Während sich für die Pegel mittellauter Hörempfindung keine signifikanten Gruppenunterschiede ergeben, zeigen sich an der Hörschwelle signifikante bis hochsignifikante Unterschiede zwischen den beiden Gruppen. Diese Unterschiede der schwellennahen Hörempfindung sind nur im Frequenzbereich von 1–4 kHz signifikant.

### Sprachverständlichkeit in Ruhe

**Figure 3:**
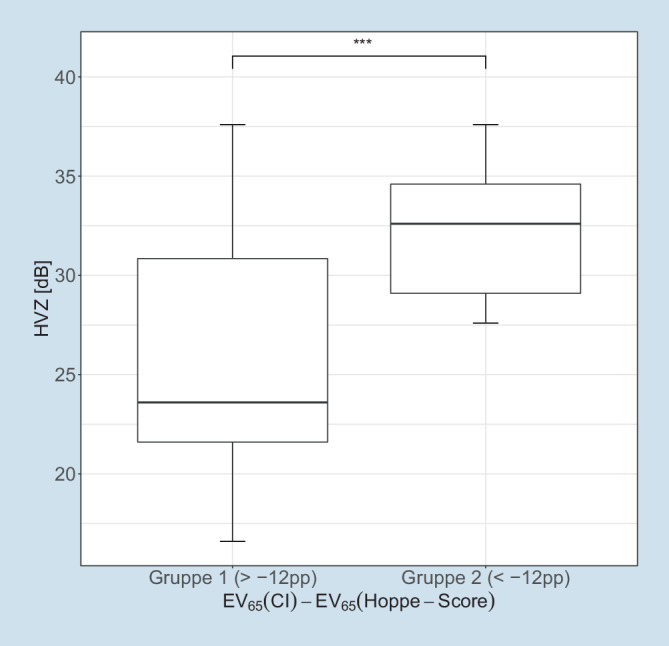

Die gruppierte Darstellung des HVZ mit CI nach 6 Monaten Versorgungsdauer zeigt Abb. 3. Die Gruppen weisen einen hochsignifikanten Unterschied des HVZ und eine größere Streubreite der Messergebnisse für Gruppe 1 auf.

**Figure 4:**
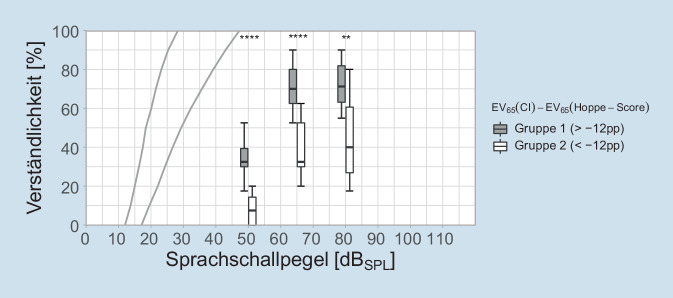

Die Einsilberverständlichkeit mit CI nach 6 Monaten Versorgungsdauer sind in Abb. 4 ebenfalls gruppiert dargestellt. Durch die Gruppenzuordnung erweist sich trivialerweise der Gruppenunterschied für EV_65_(CI) als signifikant. Dieser Unterschied zwischen Gruppe 1 und Gruppe 2 zeigt sich ebenso bei den flankierenden Sprachschallpegeln, allerdings auf anderem Signifikanzniveau.

### Sprachverständlichkeit im Störschall

**Figure 5:**
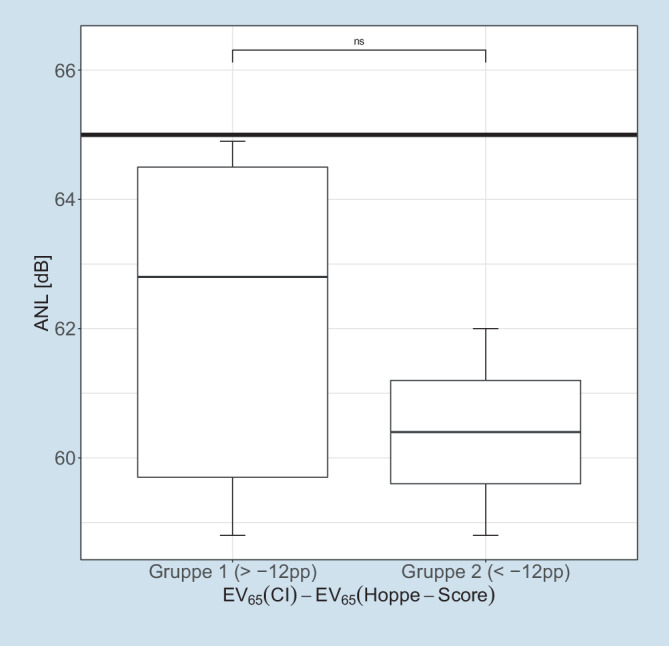

Die in Abb. 5 dargestellte Sprachverständlichkeit im Störschall vervollständigt die in den vorangegangenen Abbildungen beschriebenen Ergebnisse der postoperativen Audiometrie mit CI.

Aufgetragen ist hier der ANL nach Dziemba et al. [9] für die beiden Gruppen als Lage- und Streumaß. Für diese Messung finden sich keine signifikanten Unterschiede zwischen den beiden Gruppen.

### Erweiterung des GLM

Zur Erklärung der festgestellten Abweichungen der gemessenen 6‑Monats-Werte für das Einsilberverstehen von der Prognose (Gl. 1) wurde das bestehende GLM um folgende postoperativ gemessene Größen erweitert: den HVZ und den prozentualen Hörverlust, berechnet aus der Hörfeldskalierung. Die stark überschwelligen Messwerte aus der Hörfeldskalierung für 25 CU wurden nicht in das Modell integriert (*p* = 0,52). Somit ergibt sich Gl. 2 zu

2 $$\mathrm{EV}_{65}\left(\mathrm{CI}\right)\left[\mathrm{{\%}}\right]=\frac{100}{1+e^{-\left(\beta _{0}+\beta _{1}\cdot \mathrm{mEV}+\beta _{2}\cdot \text{Alter}+\beta _{3}\cdot \mathrm{EV}_{65}(\mathrm{HG}){+\gamma _{0}}+\gamma _{1}\cdot pHV+\gamma _{2}\cdot HVZ\right)}}$$

mit den in Tab. 3 aufgeführten Faktoren γ.

**Table 3:** 

		Wert	Standardabweichung	t‑Statistik	*p*-Wert
Konstante	γ_0_	2,98	±0,47	6,33	2,47e^−10^
pHV_Rader_	γ_1_	−0,031	±0,0096	−3,27	0,0011
HVZ	γ_2_	−0,070	±0,014	−5,06	4,26e^−07^

*HVZ *Hörverlust für Zahlwörter, *pHV* prozentualer Hörverlust

**Figure 6:**
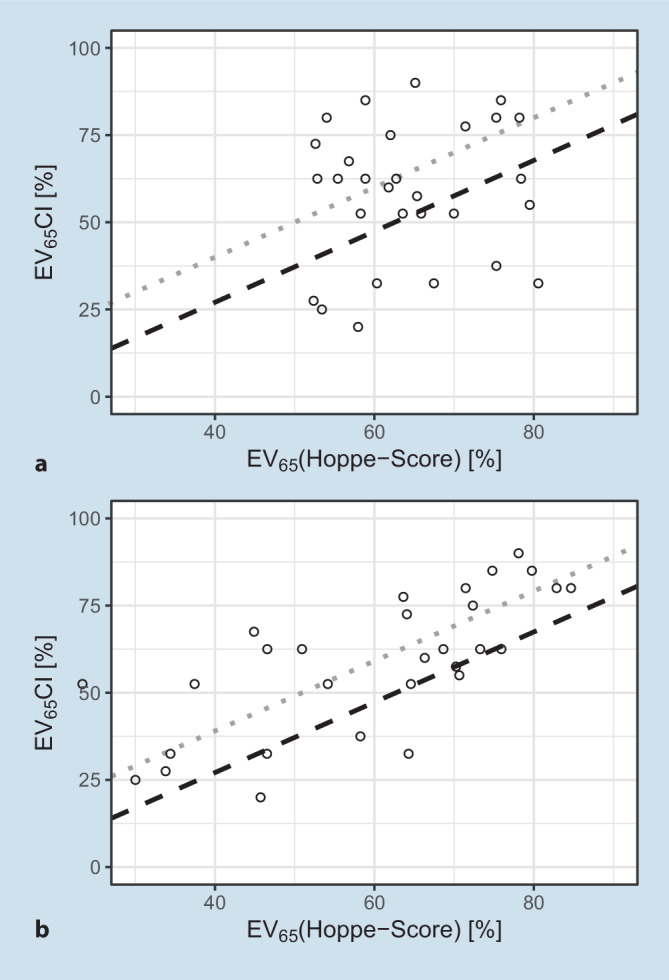

In Abb. 6 sind die EV_65_(CI), 6 Monate postoperativ, jeweils über der prognostizierten Sprachverständlichkeit nach Gl. 1 (Abb. 6a) bzw. Gl. 2 (Abb. 6b) dargestellt. Während die in Abb. 6a dargestellten Daten nicht korrelieren (R_Spearman_ = 0,098; *p* = 0,61), ergab sich unter Hinzunahme oben angeführter anpassungsbezogener Werte ein signifikanter Zusammenhang (R_Spearman_ = 0,74; *p* = 4·10^−6^). Die in Abb. 6a sichtbare große Variabilität lässt sich nun zu 55 % durch prinzipiell beeinflussbare Einstellungen des CI-Systems erklären.

## Diskussion

In der vorliegenden Studie wurde ein Modell zur Vorhersage der Sprachverständlichkeit nach Cochlea-Implantation auf eine ausgewählte Population von CI-Träger*innen angewendet. Patient*innen mit potenziell negativem Einfluss der Ätiologie auf das Versorgungsergebnis (nach Blamey et al. [1]) wurden ausgeschlossen, um mögliche anpassungsbedingte Ursachen für Abweichungen von der prognostizierten Sprachverständlichkeit zu untersuchen.

Es wurde gezeigt, dass die Variabilität der Ergebnisse ihre Ursache zu einem beträchtlichen Teil in potenziell optimierbarer Einstellung der CI-Systeme, besonders im Bereich schwellennaher Lautheit, findet.

In den 6‑Monats-Daten findet sich keine Korrelation zwischen vorhergesagter und gemessener Einsilberverständlichkeit, Abb. 1a. Das wichtige Ergebnis ist hier, dass sich durch Hinzunahme einfacher Daten aus der audiometrischen Evaluation 55 % der Variabilität der Versorgungsergebnisse erklären lassen. Das GLM aus Gl. 1 wurde auf diese Weise von einem Vorhersagemodell zu einem Erklärungsmodell nach Gl. 2 transformiert. Der signifikante Einfluss der postoperativ gemessenen Größen, hier HVZ und Kurven gleicher Lautheitskategorie, bzw. der nichtsignifikante Einfluss anderer Größen auf die erreichte Einsilberverständlichkeit birgt jedoch einen möglichen Fehlschluss. In einem Modell, welches post hoc die Variabilität der Ergebnisse „erklären muss“, gibt es 2 Möglichkeiten der Interpretation. Sobald ein Faktor, wie etwa ein Anpass-Parameter, nahezu identisch in der untersuchten Population auftritt, kann selbiger zu keinem signifikanten Testergebnis für die Erklärung der Variabilität führen. Diese Eigenschaft der Analyse mittels GLM ist nicht gleichzusetzen mit einem Bedeutungsverlust eben dieses Faktors. So würde dann erst eine nicht mehr optimale Einstellung eines bestimmten Faktors die entsprechende Variabilität in den Ergebnissen verursachen und somit vom erweiterten GLM als signifikante Einflussgröße identifiziert werden. In dieser Untersuchung hatte z. B. die eingestellte Dynamik des CI-Systems laut erweitertem GLM keinen signifikanten Einfluss auf die Einsilberverständlichkeit. Augenscheinlich erklären die geringen Abweichungen in der mittellaut empfundenen Kategorie (25 CU) die gefundenen Unterschiede im Sprachverstehen nicht, entweder aufgrund einer hinreichend guten diesbezüglichen Einstellung der Systeme oder aufgrund unzureichender Fallzahlen. So zeigen die schwellennahen Ergebnisse der Hörfeldskalierung in Abb. 2 in Gruppe 2 über den gesamten Frequenzbereich, dass erst bei zu hohen Pegeln eine Hörbarkeit erreicht wird und sich diese im Bereich 1–4 kHz auch signifikant von Gruppe 1 unterscheidet. Bei den Ergebnissen der mittellauten Hörfeldskalierung ist das Bild hingegen uneinheitlich. In Gruppe 2 wird eine Lautheit von 25 CU in den tiefen Frequenzen bei geringeren Pegeln als in Gruppe 1 erreicht, während die Lautheit von 25 CU in den hohen Frequenzen erst bei höheren Pegeln erreicht wird. Eine mögliche Interpretation wäre, dass die in der CI-Anpassung abgefragte Gesamtlautheit bei Gruppe 2 überwiegend durch die tieffrequenten Signalanteile erreicht wird, hingegen die hochfrequenten Anteile in Gruppe 2 tendenziell weniger zur Gesamtlautheit beitragen als in Gruppe 1. In Anbetracht des Frequenzgehalts der informationstragenden Konsonanten ist dieser Befund eine potenzielle Erklärung für das unterschiedliche Sprachverstehen von Gruppe 1 und 2. Allein die zu geringen Fallzahlen lassen hier keine belastbare Aussage zu. Zukünftige Studien in einer größeren Population sind eine Möglichkeit, diese eventuell systematischen Gründe für niedrigeres Sprachverstehen zu bestätigen bzw. auszuschließen.

Ein GLM der hier beschriebenen Art lässt sich im Rahmen der CI-Versorgung unterschiedlich einsetzen. zum einen für die Vorhersage des Versorgungsergebnisses, zum anderen im Rahmen der postoperativen Qualitätssicherung. Ersteres beruht auf präoperativ messbaren Einflussfaktoren, die eine gewisse Allgemeingültigkeit haben, und somit auf Patientenpopulationen verschiedener Einrichtungen und nach entsprechender Adaptation auch Länder anwendbar sind. Die zweite, hier vorgestellte Anwendung beschränkt sich zunächst in ihrer Gültigkeit auf Prozesse innerhalb einer Einrichtung oder u. U. auf eine spezielle Population. Prozessbedingte Abweichungen vom vorhergesagten Ergebnis bzw. deren Ursachen könnten durchaus nur auf einzelne Einrichtungen zutreffen. Es wäre auch denkbar, dass eine CI-Population mit eng umschriebenen, speziellen Eigenschaften (z. B. Hyperakusis, Tinnitus oder unzureichende Compliance) andere erklärende Faktoren herausstellt. Das aus den vorliegenden Daten abgeleitete GLM bietet eine Möglichkeit, systematische Ursachen für unterschrittene Vorhersagewerte zu erkennen und entsprechende multidisziplinäre Maßnahmen im Rahmen der CI-Folgetherapie einzuleiten. Diese Maßnahmen waren nicht Teil dieser Beobachtungsstudie, bieten jedoch begründete, systematische (!) Ansätze zur Verbesserung der Versorgungsqualität. Der Ausschluss präoperativ nicht abzusehender Einschränkungen des Versorgungsergebnisses durch (noch) nicht diagnostizierbare retrocochleäre Hörstörungen sollte zukünftig durch geeignete Messmethoden von reinen Anpassungsdefiziten getrennt werden. Eine Möglichkeit stellt die objektive Hörbahndiagnostik mittels elektrophysiologischer Methoden dar [10, 18, 19].

## Fazit für die Praxis

- Das hier beschriebene Modell ist geeignet, um in einer umschriebenen Population mit Cochlea-Implantat (CI) einzelne, die Sprachverständlichkeit mindernde Faktoren innerhalb der CI-Folgetherapie zu identifizieren.
- Die Ergebnisse zeigen, dass sich aus der postoperativen Audiometrie mit CI direkte Rückschlüsse für die Optimierung der individuellen CI-Anpassung ziehen lassen.
